# Advances in Nanostructured Electrode Materials: Design and Applications

**DOI:** 10.3390/nano15211638

**Published:** 2025-10-27

**Authors:** Maria Grazia Musolino

**Affiliations:** 1 Department of Civil, Energy, Environmental and Materials Engineering (DICEAM), Mediterranean University of Reggio Calabria, 89122 Reggio Calabria, Italy; mariagrazia.musolino@unirc.it; 2 National Reference Center for Electrochemical Energy Storage (GISEL), National Interuniversity Consortium for the Science and Technology of Materials (INSTM), 50121 Florence, Italy

The development of nanostructured electrode materials is a cornerstone of emerging electrochemical technologies that provides clean and sustainable solutions to address the global energy demand and the rapid depletion of fossil fuels, as well as environmental pollution issues such as global warming, climate change, and air/water pollution resulting from the overexploitation of carbon-based energy resources. The unique and outstanding features of these materials, arising from the ability to suitably tailor their structural and functional properties, are key to optimizing performance, durability, and efficiency. However, the design, synthesis, and characterization of nanostructured electrodes remain a current area of research, facing challenges such as scalable fabrication and structural stability.

The Special Issue of *Nanomaterials* “Advances in Nanostructured Electrode Materials: Design and Applications” provides an overview of the current state of the art in the field of nanostructured electrode materials, including their synthesis, properties, characterization, and applications in electrocatalysis, energy conversion, energy storage, and environmental protection. It especially promotes the interdisciplinary aspects of materials science and the interactions between fundamental research and technology. This Special Issue collects eight original research papers, summarized below, covering a wide range of electrode materials and their technological applications.

Cai et al. investigated the effects of introducing elemental tungsten (W) into GeTe-based materials on the material structure and electrical and thermal transport properties [1]. Experimental data indicated that the increase in electrical conductivity of the sample was due to the presence of high-valence state W atoms, additional charge carriers, thus improving the Seebeck coefficient. Furthermore, appropriate W doping concentrations reduced the lattice thermal conductivity by optimizing the material structure, thus comprehensively tuning the thermoelectric transport properties of GeTe systems.

Conti et al. synthesized a self-standing electrode for sodium-ion batteries (SiBs), based on a Na_3_MnTi(PO_4_)_3_ active material loaded into carbon nanofibers (CNFs), by electrospinning [2]. The active material was homogeneously spread into CNFs and displayed a NASICON-type crystal structure, but the high sintering temperature (750 °C) used to obtain conductive CNFs induced cell shrinkage, thus implying a sluggish redox activity. This study highlighted the promising electrochemical performances of this electrode compared to its conventional tape-casted counterpart, thanks to the easy electrolyte diffusion and contact with the active material afforded by the porous nature of non-woven nanofibers.

The work of Beitia et al. focused on the synthesis, characterization, and electrochemical performance of zinc-doped manganese hexacyanoferrate systems, used as cathodes for aqueous Zn-ion batteries [3]. Zinc doping was a useful strategy to improve the weak structural stability of manganese hexacyanoferrate (MnHCF) in an aqueous environment and reduce manganese dissolution. Despite a decrease in the specific capacity of the system, Mn_1−x_Zn_x_HCF (x = 0, 0.25, 0.5, 0.75 and 1) Prussian Blue analogues, prepared through a simple and easy-to-implement approach, improved the stability and capacity retention of the cathode. Furthermore, the amount of zinc introduced into the MnHCF played a critical role in achieving higher reversibility and stable performance for the MnHCF phase without a drastic loss in capacity.

A novel design concept, where paper can act as both a separator and a substrate for coating the anode material for lithium-ion batteries, is reported in the work of Blomquist et al. [4]. A fully disposable and resource-efficient paper-based electrode was successfully fabricated via large-scale roll-to-roll coating technology, where the conductive material is a nanographite and microcrystalline cellulose mixture coated on a paper separator. The produced electrode material, tested in a typical lithium ion half-cell coin cell setup, exhibited a specific capacity of 147 mAh/g and a good long-term stability of the battery capacity over extended cycling.

Gasparotto et al. developed the chemical vapor infiltration (CVI) method for the one-step synthesis of polymeric carbon nitride (PCN) films on porous Ni foam substrates, starting from melamine as a precursor compound [5]. By varying the reaction temperature and the precursor amount, PCN deposits with a tunable condensation degree (from melem/melon hybrids to melon-like materials) and different morphological features are obtained. PCN-based electrodes with different polymerization degrees showed promising catalytic performances in the oxygen evolution reaction (OER), considered a bottleneck of the water splitting process.

A new type of Metal–Organic Framework (MOF) material (metal-triazolates) on nickel foam (NF) substrates, synthesized by a solvothermal method, was directly used as a self-supporting electrode for the OER, enabling performances that outperform most of the reported OER catalysts [6]. Among various metal-triazolates, the Fe-based one on (MET-Fe/NF) exhibited the best OER performance, achieving a low overpotential of 122 mV at a current density of 10 mAcm^−2^ and maintaining good stability over 15 h. The experimental results showed that MET-Fe/NF underwent structural reconstruction during the OER process, resulting in a hybrid catalyst with several active components (iron/nickel (oxy)hydroxides) with high OER activity. Furthermore, in a two-electrode water splitting setup, this electrocatalyst, used as an anode, displayed a good performance, allowing for continuous hydrogen and oxygen generation at a low voltage of 1.46 V.

Sako et al. fabricated Ni nanowire array electrodes with an extremely large surface area by a potentiostatic electrodeposition technique into anodized alumina nanochannels [7]. The electrodeposited Ni nanowire arrays had a textured structure with a preferential orientation in the fcc-Ni (111) plane, regardless of the electrodeposition potential and exhibited uniaxial magnetic anisotropy, with easy magnetization in the axial direction. The electrocatalysts exhibited a lower overpotential and a higher current density towards the hydrogen evolution reaction compared to electrodeposited Ni films.

Campagna Zignani et al. proposed a very simple and scalable process for the synthesis of low cost and efficient bimetallic oxide-based electrocatalysts for “green hydrogen” production from water electrolysis [8]. Nanostructured NiCo- and NiFe-based electrode materials with different Ni molar fractions were prepared by the sol–gel method and subsequent calcination in air at different temperatures, and then evaluated as anode materials in a zero-gap anion exchange membrane water electrolysis (AEMWE) full cell. The cell cathodes were fabricated using the same materials after reduction in a H_2_/Ar atmosphere. The electrochemical results revealed that the nanomaterial phase purity and the average crystal size were critical in determining cell performance. Highly pure and finely grained electrocatalysts yielded higher current densities at lower overpotentials, paving the way for scalable and cost-effective green hydrogen production from water electrolysis.

In conclusion, the research presented in this Special Issue underscores the immense potential of nanostructured electrode materials in wide range of applications. Each paper provides valuable insights into the synthesis, characterization, and application of these materials, showcasing their versatility. Although this Special Issue cannot fully cover the topic of electrode materials, I firmly believe that its contributions will open new perspectives in the field of materials and technologies, promoting the development of advanced nanomaterials as electrodes, combining low-cost, sustainable materials with simple manufacturing processes, in line with future clean energy goals.
